# Inhibition of Rho Kinase by Fasudil Ameliorates Cognition Impairment in APP/PS1 Transgenic Mice via Modulation of Gut Microbiota and Metabolites

**DOI:** 10.3389/fnagi.2021.755164

**Published:** 2021-10-14

**Authors:** Yuqing Yan, Ye Gao, Qingli Fang, Nianping Zhang, Gajendra Kumar, Hailong Yan, Lijuan Song, Jiehui Li, Yuna Zhang, Jingxian Sun, Jiawei Wang, Linhu Zhao, Keith Skaggs, Han-Ting Zhang, Cun-Gen Ma

**Affiliations:** ^1^Shanxi Key Laboratory of Inflammatory Neurodegenerative Diseases, Institute of Brain Science, Medical School of Shanxi Datong University, Datong, China; ^2^The Key Research Laboratory of Benefiting Qi for Acting Blood Circulation Method to Treat Multiple Sclerosis of State Administration of Traditional Chinese Medicine, Research Center of Neurobiology, Shanxi University of Chinese Medicine, Taiyuan, China; ^3^Department of Neuroscience, City University of Hong Kong, Hong Kong, Hong Kong, SAR China; ^4^McGovern Institute for Brain Research, Massachusetts Institute of Technology, Cambridge, MA, United States; ^5^Department of Pharmacology, Qingdao University School of Pharmacy, Qingdao, China

**Keywords:** Alzheimer's disease, metagenomics, metabolite, cognition, gut microbiota, Morris water maze, APP/PS1 double transgenic AD mouse

## Abstract

**Background:** Fasudil, a Rho kinase inhibitor, exerts therapeutic effects in a mouse model of Alzheimer's disease (AD), a chronic neurodegenerative disease with progressive loss of memory. However, the mechanisms remain unclear. In addition, the gut microbiota and its metabolites have been implicated in AD.

**Methods:** We examined the effect of fasudil on learning and memory using the Morris water-maze (MWM) test in APPswe/PSEN1dE9 transgenic (APP/PS1) mice (8 months old) treated (i.p.) with fasudil (25 mg/kg/day; ADF) or saline (ADNS) and in age- and gender-matched wild-type (WT) mice. Fecal metagenomics and metabolites were performed to identify novel biomarkers of AD and elucidate the mechanisms of fasudil induced beneficial effects in AD mice.

**Results:** The MWM test showed significant improvement of spatial memory in APP/PS1 mice treated with fasudil as compared to ADNS. The metagenomic analysis revealed the abundance of the dominant phyla in all the three groups, including *Bacteroidetes* (23.7–44%) and *Firmicutes* (6.4–26.6%), and the increased relative abundance ratio of *Firmicutes*/*Bacteroidetes* in ADNS (59.1%) compared to WT (31.7%). In contrast, the *Firmicutes*/*Bacteroidetes* ratio was decreased to the WT level in ADF (32.8%). Lefse analysis of metagenomics identified *s_Prevotella_sp_CAG873* as an ADF potential biomarker, while *s_Helicobacter_typhlonius* and *s_Helicobacter_sp_MIT_03-1616* as ADNS potential biomarkers. Metabolite analysis revealed the increment of various metabolites, including glutamate, hypoxanthine, thymine, hexanoyl-CoA, and leukotriene, which were relative to ADNS or ADF microbiota potential biomarkers and mainly involved in the metabolism of nucleotide, lipids and sugars, and the inflammatory pathway.

**Conclusions:** Memory deficit in APP/PS1 mice was correlated with the gut microbiome and metabolite status. Fasudil reversed the abnormal gut microbiota and subsequently regulated the related metabolisms to normal in the AD mice. It is believed that fasudil can be a novel strategy for the treatment of AD via remodeling of the gut microbiota and metabolites. The novel results also provide valuable references for the use of gut microbiota and metabolites as diagnostic biomarkers and/or therapeutic targets in clinical studies of AD.

## Introduction

Alzheimer's disease (AD) is a complex heterogenous disease characterized by progressive impairment of memory and cognition and most prevalent cause of dementia (Fan et al., 2019). Decreased expression of amyloid β (Aβ) peptides and phosphorylated Tau (pTau) protein are assumed to lower the probability of developing AD (Mantzavinos and Alexiou, 2017). AD has emerged as a severe medical concern worldwide due to a lack of sensitive, accurate, and accessible biomarkers for detection, diagnosis, and monitoring of the disease progression (Mietelska-Porowska and Wojda, 2017; Mila-Aloma et al., 2019). Therefore, it is of the utmost importance to develop the most suitable biomarkers for AD. Recently, the human gut microbiota has been reported to play a critical role in the maintenance of normal physiological condition and health. It has also been demonstrated to modulate the physiological aspects of neurodegenerative disorders (Fujii et al., 2019). A plethora of evidence suggests that the role of gut microbiota and its metabolites in pathogenesis and progression of AD (Minter et al., 2017; Yue et al., 2019; Marizzoni et al., 2020).

The gastrointestinal (GI) tract has 10^13^-10^14^ microorganisms, most of which belong to the Bacteroidetes and Firmicutes phyla (Cryan and Dinan, 2012). Clinical studies have shown that Bacteroides in AD patients are significantly decreased, suggesting its crucial role in health protection by providing intestinal barrier and blocking gut leakiness (Hsiao et al., 2013; Zhuang et al., 2018). Firmicutes are also known to increase as a risk factor in AD pathogenesis (Mancuso and Santangelo, 2018). The association between Prevotella and Helicobacter pylori in AD patients has been reported recently (Beydoun et al., 2020), where Helicobacter pylori was significantly increased at the genus level, while Prevotella was significantly lower in APP/PS1 mice relative to WT controls (Shen et al., 2017). Curcumin treatment has been shown to significantly change the abundance of Bacteroidaceae, Prevotellaceae and 8 metabolites in AD mice (Sun et al., 2020).

Clinical studies have demonstrated that elderly patients with dementia exhibit Aβ plagues with bacterial accumulation in the pathogenesis of cognitive impairment (Cattaneo et al., 2017; Bulgart et al., 2020; Goyal et al., 2021). Helicobacter pylori has been shown to induce Tau hyperphosphorylation in several AD-related Tau phosphorylation sites (Wang et al., 2015). Accumulating data indicate that gut microbiota communicates with CNS through the neural, endocrine, and immune pathways, thereby influencing brain function and behaviors (Cryan and Dinan, 2012). Altered gut microbial composition is associated with fecal metabolome changes (Zheng et al., 2019). Metagenomics is a new method to study microbial diversity by considering all the genomes of the microbial population in samples, which can help identify novel biomarkers (Dunn et al., 2011; Li et al., 2015; Truong et al., 2015; Huson et al., 2016).

APPswe/PSEN1dE9 transgenic (APP/PS1) mice express human amyloid precursor protein (HuAPP695swe) and a mutant human presenilin 1 (PS1-dE9) are commonly used model of AD. It has been shown that APP/PS1 mice exhibit learning and memory impairment at the age of 8 months (Li et al., 2016) and significant Aβ plaque accumulation in hippocampus at 9 months (Jankowsky et al., 2004; Yu et al., 2018). Our previous studies have demonstrated that fasudil, a selective Rho kinase (ROCK) inhibitor (Yan et al., 2019), decreases expression of ROCK-II in experimental autoimmune encephalomyelitis (EAE) mice (Yu et al., 2010). Inhibition of ROCK by fasudil reverses Aβ_1−42_-induced neuronal apoptosis, intracellular calcium overload, and decreases the mitochondrial membrane potential. Thus, ROCK inhibitors such as fasudil can be conferred as future therapeutic and preventive strategies for neuroinflammatory and neurodegenerative diseases (Gao et al., 2019). However, it has not been reported that alteration of gut microbiota by fasudil exhibit cognition-enhancing effect in AD mice. In the present study, we deciphered the close connection between the gut microbiota and AD through the microbiota-gut-brain axis. Gut microbiota could serve as a potential new target for therapeutic intervention in AD (Zhou et al., 2019; Zhu et al., 2021). The brain-gut axis controls the interaction of biochemical molecules of brain and gut (Lu et al., 2019). Therefore, we proposed that the gut microbiota and its metabolites trigger the neurodegenerative disorder through the brain-gut axis, which may be a potential mechanism of AD pathogenesis and preventive effect of fasudil in AD.

## Materials and Methods

### Animals and Treatment

Male APP /PS1 mice on the C57BL/6 background (8 months old) were purchased from Beijing Huafukang Bioscience CO., LTD (HFK, Beijing, China). Animals were housed in pathogen-free facilities at Institute of Brain Science, Shanxi Datong University and maintained constant room temperature (25 ± 2°C) and humidity (50 ± 5%) in a 12-h light/12-h dark cycle. APP/PS1 mice were pre-screened based on the normal physiological behavior and randomly divided into two treatment groups: (1) vehicle-treated mice (ADNS), which were administered normal saline (the volume was adjusted similar to fasudil treatment); (2) fasudil-treated (ADF) mice, which received a daily injection of fasudil (Tianjin Chase Sun Pharmaceutical Co., Ltd.), 25 mg/kg/day, i.p., 16 wk; age- and gender-matched C57BL/6 (WT) mice were used as normal controls, which received saline in the same volume (*n* = 7 per group). Animals had *ad libitum* access to food and water. All the experiments were performed in compliance with the guidelines and regulations of the Administration Office of the International Council for Laboratory Animal Science. The experimental protocols were approved by the Animal Ethics Committee of Shanxi Datong University, Datong, China.

### The Mouse Cognition Test

The Morris water-maze (MWM) test was used to measure the spatial learning and memory abilities of APP/PS1 and WT mice. It was performed with a pool (140 cm in diameter) filled with water, colorant (titanium dioxide) was mixed to make the platform invisible and water level was 1.5 cm above the platform. Water was maintained at a constant temperature (25 ± 2°C). The MWM apparatus was surrounded by a blue curtain with optimum light in a fixed position. MWM was divided into four quadrants; northeast (NE), northwest (NW), southeast (SE), and southwest (SW). MWM test was performed on a week after the last treatment. During the 5-day acquisition training, mice were individually trained to locate the hidden platform from the starting point for 4 trials/day with the cut-off time 60 s. On each trial, the mice were placed in the water at different start locations (E, S, W, and N), which were equally spaced from each other. The animal was allowed to locate the hidden platform by swimming for 60 s and to remain on the platform for at least 10 s. If the mouse was unable to locate the platform within 60 s, it was gently guided to the platform by the experimenter and allowed to remain on it for 10 s. In this case, its performance score (or escape latency) was marked as 60 s. The probe trial was performed on day 6 (24 h after the last training trial) when the platform was removed, and the mouse was allowed to swim freely for 60 s. The position of the mice was tracked by a camera above the center of the pool and was connected to an automatic photographic recording and analysis system (SMART V3.0 system, Panlab, Barcelona, Spain). The escape latency (i.e., the time spent in locating the hidden platform), latency of the first entrance to the target zone, and the time spent in the target zone (% of the total time in all the four zones) during the 5-d acquisition training, the swimming paths, and the number of crossings into the target quadrant during the probe trial were all recorded using the SMART V3.0 system. Experimental timeline are shown in Supplementary Figure 1 (Yu et al., 2018).

### Bielschowsky Silver-Plated Nerve Staining

The silver glycinate staining was performed following the procedures described previously (Connolly et al., 1987; Suenaga et al., 1990; Litchfield and Nagy, 2001; Cerri and Sasso-Cerri, 2003). In brief, after dehydrating with ethanol and staining with acidic formaldehyde, brain slices (pre-heated at 37°C) were placed in the silver glycine solution for 3–5 min, followed by clearing and mounting to reduce solution staining and dehydration. Microscopic examination, image acquisition, and analysis were performed (Uchihara, 2007).

### Metagenomics Sample Preparation and Analysis

All fecal samples were frozen at −80°C before DNA extraction and analysis. Fecal DNA was extracted using PowerSoil DNA Isolation Kits (MoBio Laboratories, Carlsbad, CA) following the instructions provided by the manufacturer. The purity and integrity of DNA were analyzed by agarose gel electrophoresis (AGE), and the accurate quantification of DNA concentrations was analyzed by Qubit. The DNA was sheared to 300 bp with the Covaris ultrasonic crusher. For sequencing library preparation, the fragments were treated by end repair, a tailing, and ligation of Illumina compatible adapters. After the construction, the library was initially quantified by Qubit 2.0, diluted to 2 ng/ul, and then the insert size of the library was detected by Agilent 2100. After the insert size met the expectation, the effective concentration of the library was accurately quantified by q-PCR (the effective concentration of the library was > 3 nM) to ensure the library quality. DNA sequencing libraries were deeply sequenced on the Illumina Hiseq platform at Allwegene Company (Beijing, China). The software MEGAHIT (v1.0.6) was used to assemble the sequencing samples, and the fragments below 500 bp in the assembly results were filtered out. Contigs were annotated with the Prodigal software to predict open reading frames (ORFs), and the CD-HIT software was used to construct the non-redundant gene set. Bowtie was used to compare the sequencing data with the non-redundant gene set, and the abundance information of genes in different samples was counted.

The high-quality sequences were compared with the NR database (i.e., the non-redundant protein sequence database) and classified into different taxonomic groups by the diamond BLAST tool. Clustering analysis and principal component analysis (PCA) were used to examine the similarities between different samples, based on the genus information from each sample. The gene function was annotated by and searched against the functional annotation database KEGG, GO and CAZyme.

### Metabolomic and Metabolites Analysis

The metabolites were detected by an ultra-high-performance liquid tandem chromatography quadrupole time of flight mass spectrometry (UHPLC-QTOFMS) at the Allwegene Company (Beijing, China), including basic and personalized data analyses (Su et al., 2020). The data statistical analysis, which includes data preprocessing, Student's *t*-test, PCA, and differential metabolite screening, was carried out by MetaX on the qualitative data of the metabolome for screening metabolites with significant differences. Generally, the threshold with *p* < 0.05 and VIP (Variable Importance in the Projection) > 1 was used to screen differential metabolites. The VIP values exceeding 1.0 were first selected as changed metabolites; the remaining variables were then assessed by Student's *t*-test (*p* < 0.05). Personalized analyses included the KEGG analysis and metabolic pathway analysis of differential metabolites. Commercial database including KEGG (http://www.kegg.jp) and MetaboAnalyst (http://www.metaboanalyst.ca/) were utilized to search for the pathways of metabolites.

### Association Analysis of the Metagenomic and Metabolomic

The Spearman correlation coefficient was calculated based on features with in the metagenomic and metabolomic data, and R language GGPlot package was used to draw correlation heat map.

### Statistical Analysis

The SPSS software (International Business Machines Corporation, IBM, USA) was used for statistical analysis. All data were expressed as means ± SEM. Differences among multiple groups were analyzed by one-way analysis of variance (ANOVA), with *post hoc* comparisons of Dunnett tests. *t*-test was used to compare two groups. A value of ^*^p < 0.05, ^**^ *p* < 0.01, ^#^*p* < 0.05, ^##^*p* < 0.01 was considered statistically significant. Graphical presentations were performed with GraphPad Prism (version 8.0) software (GraphPad 122 Software, San Diego, IL, USA).

## Results

### Fasudil Improved Cognitive Function in APP/PS1 Mice

**Figure 1:**
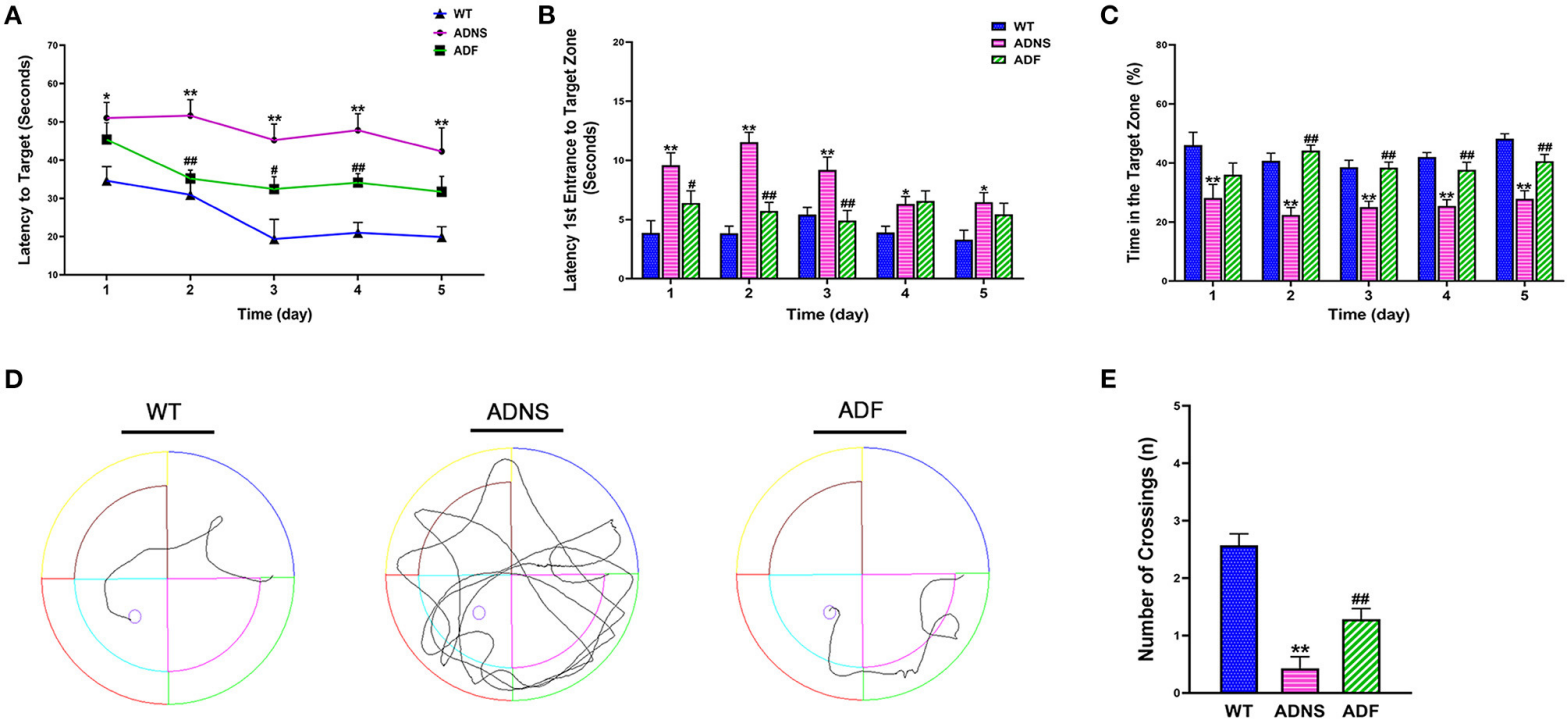
Effects of fasudil on learning and memory of APP/PS1 mice in the Morris water maze test. **(A–C)** Changes in escape latency **(A)**, latency of first entrance to the target zone **(B)**, and the total time (%) spent in target zone **(C)** during the 5-d acquisition training in WT, APP/PS1 mice treated with saline (ADNS) or fasudil (ADF). **(D)** Representative swimming paths of WT, ADNS, and ADF mice in the probe trial. **(E)** The number of crossings into the border of the target zone in the probe trial. Data presented are the means ± SEM; *n* = 7; ^*^p < 0.05, ^**^p < 0.01 vs. WT; ^#^*p* < 0.05, ^##^*p* < 0.01 vs. ADNS.

The MWM test was used to assess the effect of fasudil on cognitive function in APP/PS1 mice at the age of 12 months. During the 5-d training period, APP/PS1 mice treated with saline (ADNS) showed significant increase in the latency to locate the platform (Figure 1A), latency of the first entrance into the target zone (SW) (Figure 1B), and decreased in the time (%) spent in SW (Figure 1C) when compared with WT + saline (WT) (*p* < 0.05 or *p* < 0.01). In contrast, APP/PS1 mice treated with fasudil (ADF) showed significant decreased in the two latencies and increased in the time (%) in the target zone, as compared with ADNS (*p* < 0.05 or *p* < 0.01; Figures 1A–C). Representative raw traces of swimming in the probe trial test (Day 6) were shown in Figure 1D. The traces of ADNS mice were significantly longer than WT and shortened in ADF. Similarly, the number of crossings over the target area was significantly lower in ADNS as compared to WT, which was attenuated in ADF (*p* < 0.01; Figure 1E). These results suggest that the impairment of learning and memory in the AD mice were reversed or attenuated by treatment with fasudil.

### Fasudil Treatment Decreased Senile Plaques and Neurofibrillary Tangles in the Hippocampus of AD Mice

**Figure 2:**
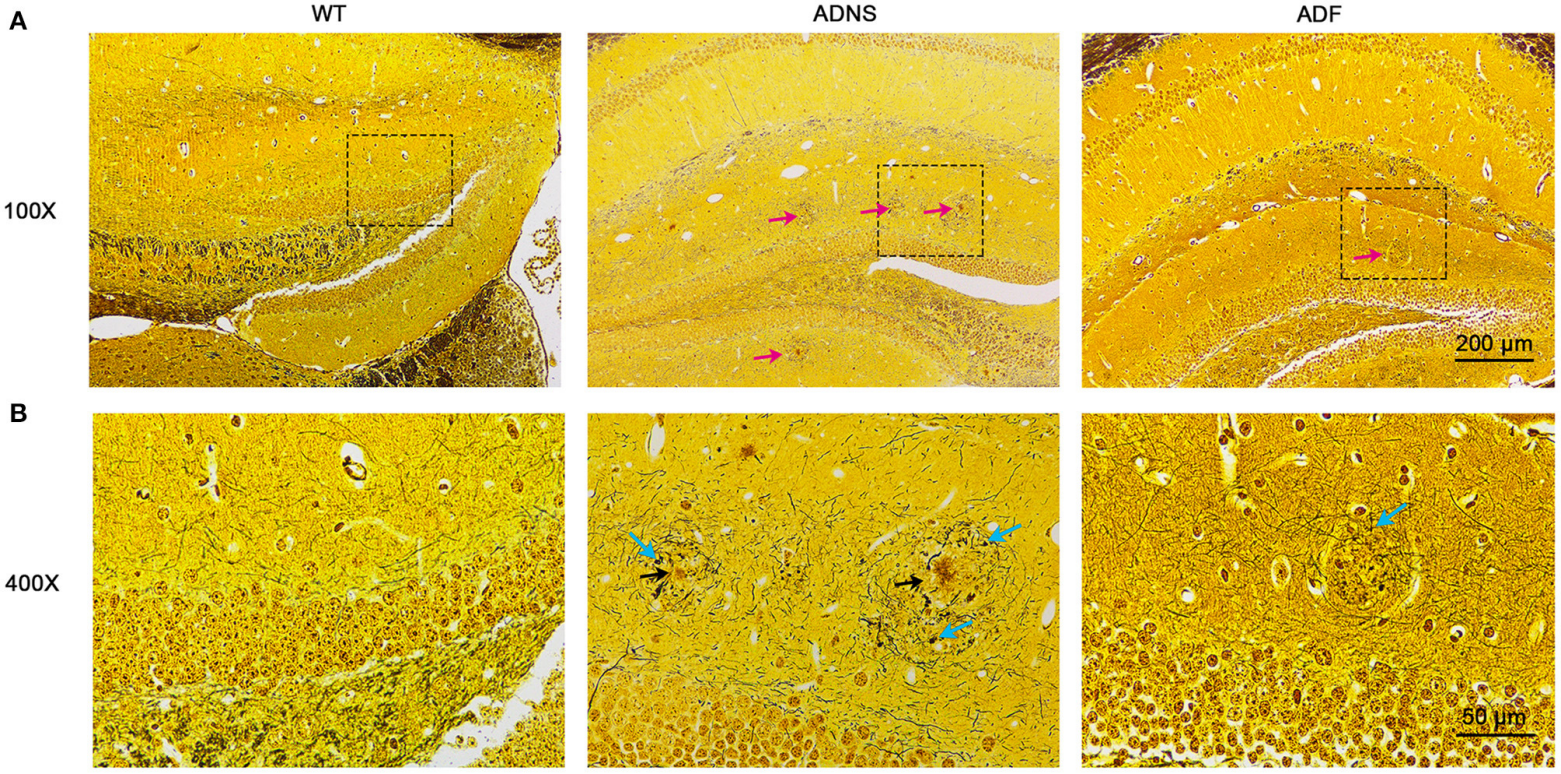
Senile plaques and neurofibrillary tangles in the mouse hippocampus with Bielschowsky silver-plated nerve staining. **(A)** Representative images of hippocampal sections from WT and APP/PS1 mice treated with saline (ADNS) or fasudil (ADF). The neurofibrillary tangles (red arrow) were densely distributed in ADNS, but not in WT and only sparsely observed in ADF. **(B)** Magnification of the representative images (squared areas) of the hippocampal sections from WT, ADNS, and ADF mice. A large number of senile plaques (black arrow) and neurofibrillary tangles (blue arrow) were visible in ADNS, but not in WT and only occasionally in ADF. Scale = 200 μm **(A)** or 50 μm **(B)**.

To identify Aβ plaques and neurofibrillary tangles in the hippocampus, we used Bielschowsky silver staining, the most commonly used method for examining senile plaques and neurofibrillary tangles (Suenaga et al., 1990; Segura-Anaya et al., 2018). It was observed that a large number of senile plaque deposition and neurofibrillary tangles were accumulated in the hippocampus of ADNS mice, but not in WT controls (Figures 2A,B). This was remarkably attenuated as both senile plaques and neurofibrillary tangles were significantly decreased in the hippocampus of ADF as compared to ADNS mice.

### Fasudil Treatment Reversed the Specific Alterations of Gut Microbial Diversity in AD Mice

**Figure 3:**
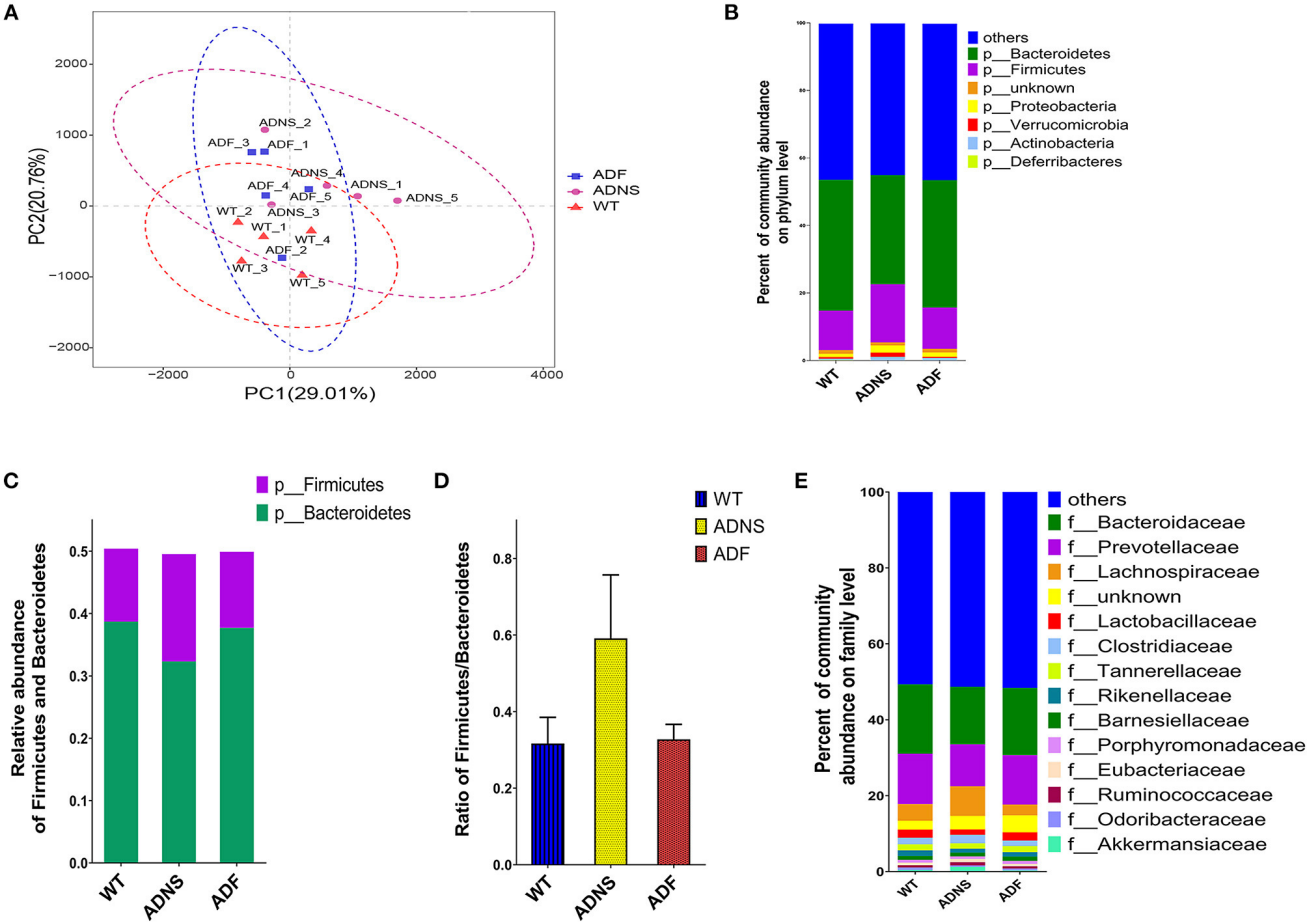
Microflora alteration in WT and APP/PS1 mice treated with fasudil. **(A)** Comparison of microbiota based on species composition by principal component analysis (PCA) in WT (red triangles) and APP/PS1 mice treated with saline (ADNS, purple circles) or fasudil (ADF, blue squares). The colored ellipses indicate 0.95 confidence interval (CI) ranges within each tested group. The scale of the horizontal and vertical axes refers to a relative distance. PC1 and PC2 represent the suspected influencing factors for the deviation of the species composition of samples. The closer the distance between the two points represents the smaller the difference of species abundance composition between the two samples. The data indicate ADF is closer to WT than ADNS. **(B)** The dominant representation in phyla. WT, ADNS, and ADF were colored at the phylum level on a stream graph. Bacteroides (green) and Firmicutes (purple) were the two most abundant bacteria at the phylum level. **(C,D)** The two most abundant phyla of Bacteroidetes and Firmicutes. The columns indicate the abundance of Bacteroidetes (green) and Firmicutes (purple) **(C)**. The ratio of Firmicutes/Bacteroidetes was increased in ADNS compared to WT, which was reversed in ADF **(D)**, indicating an alteration in the types of bacteria. **(E)** The dominant levels of bacterium families in WT, ADNS, and ADF mice, which were colored on a stream graph. The order of relative abundance is: Bacteroidaceae > Prevotellaceae > Lachnospiraceae > Lactobacteriaceae; *n* = 5.

To assess the effects of fasudil on gut microbiota alterations related to the pathogenesis of AD, we used Principal Component Analysis (PCA) by variance decomposition to reflect the differences between groups using APP/PS1 mice and WT controls. Species annotation abundance based on PCA was performed. ADNS showed different species abundance composition, while WT and ADF showed similar composition of species abundance (Figure 3A). In addition, we performed species annotated relative abundance bar chart analysis, which showed dominant phyla in all groups, including *p-Bacteroidetes* (23.7–44%) and *p-Firmicutes* (6.4–26.6%) (Figure 3B and Supplementary Table 1: the dominant phyla in all groups). In these two most dominant phyla, the relative abundance ratio of *Firmicutes/Bacteroidetes* was increased in ADNS (59.1%) as compared to WT (31.7%), while decreased in ADF (32.8%) relative to ADNS (Figures 3C,D), suggesting the progression of AD may be associated with a high proportion of *Firmicutes*/*Bacteroidetes*, which could be lowered to normal following fasudil treatment. Further, we analyzed the data at the family levels and observed that *f-Bacteroidaceae, f-Prevotellaceae, f-Lachnospiraceae*, and *f-Bacteroidaceae* were relatively the most abundant in all groups (Figure 3E and Supplementary Table 2: the level of family in all groups).

**Figure 4:**
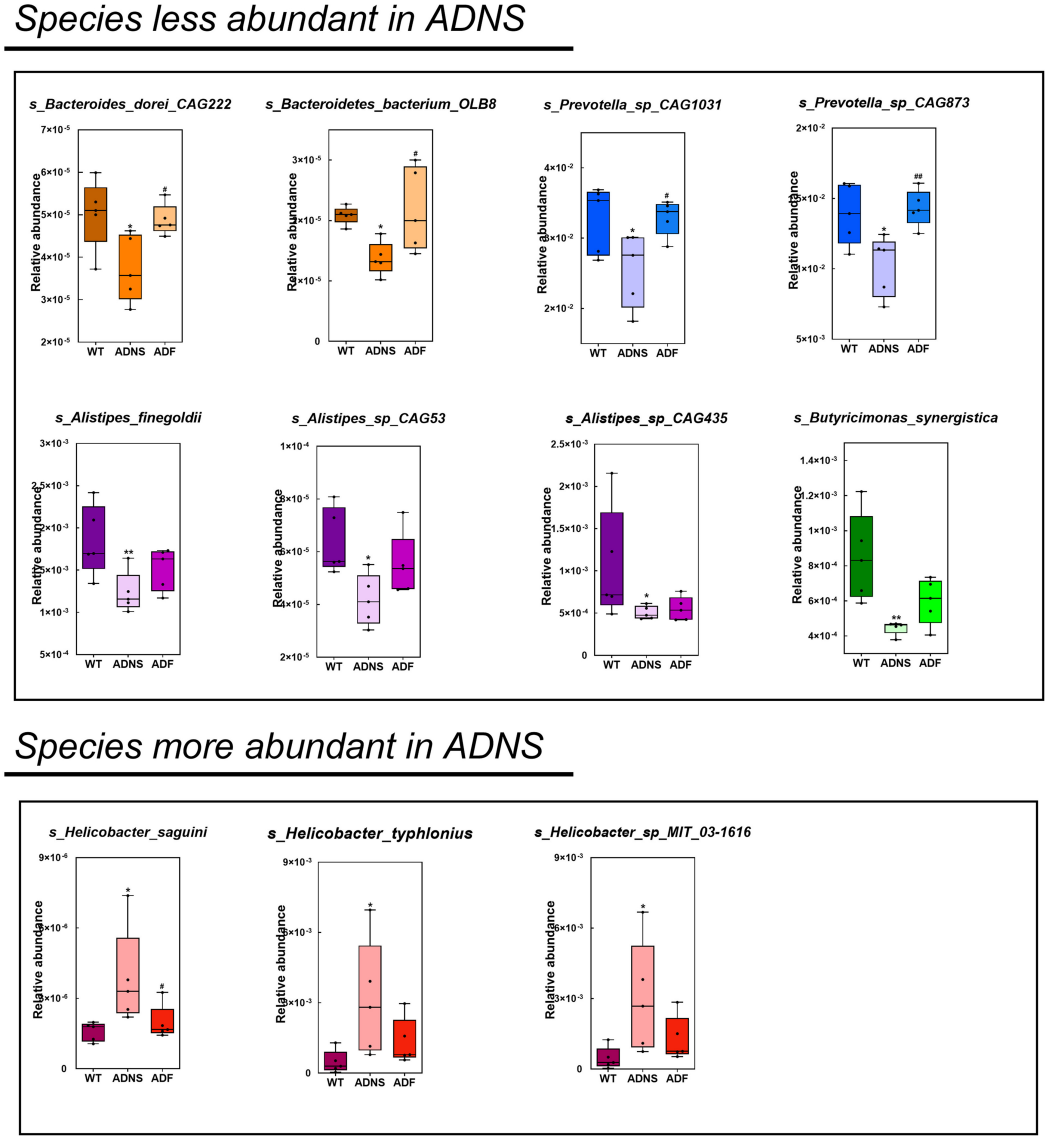
Comparison of bacterial species in WT, ADNS and ADF mice. Each box plot represents the median, interquartile range, minimum, and maximum values for the relative abundance of individual species. Species with less abundant in ADNS compared to WT (*p* < 0.05), which was reversed in ADF, included (upper): *s_Bacteroides_dorei_CAG222* (*p* < 0.05), *s_Bacteroidetes_bacterium_OLB8* (*p* < 0.05) (both in orange), *s_Prevotella_sp_CAG1031* (*p* < 0.05), and *s_Prevotella_sp_CAG873* (*p* < 0.01) (both in blue). Species with less abundant in ADNS compared to WT, which was not significantly blocked in ADF, included (middle): *s_Alistipes_finegoldii* (*p* < 0.01), *s_Alistipes_sp_CAG53* (*p* < 0.05), *s_Alistipes_sp_CAG435* (*p* < 0.05) (all in purple), and *s_Butyricimonas_synergistica* (*p* < 0.01; in green). Species with more abundant in ADNS compared to WT (*p* < 0.05), which was significantly blocked in ADF, included (lower left) *s_Helicobacter_saguini* (*p* < 0.05; in pink); species with increased abundance in ADNS relative to WT (*p* < 0.05), which was not significantly attenuated in ADF, included (lower middle and right) *g_Helicobacter* (*s_Helicobacter_typhlonius* and *s_Helicobacter_sp_MIT_03-1616*); *n* = 5. *p < 0.05, **p < 0.01 vs. WT; ^#^*p* < 0.05, ^*##*^*p* < 0.01 vs. ADNS.

**Figure 5:**
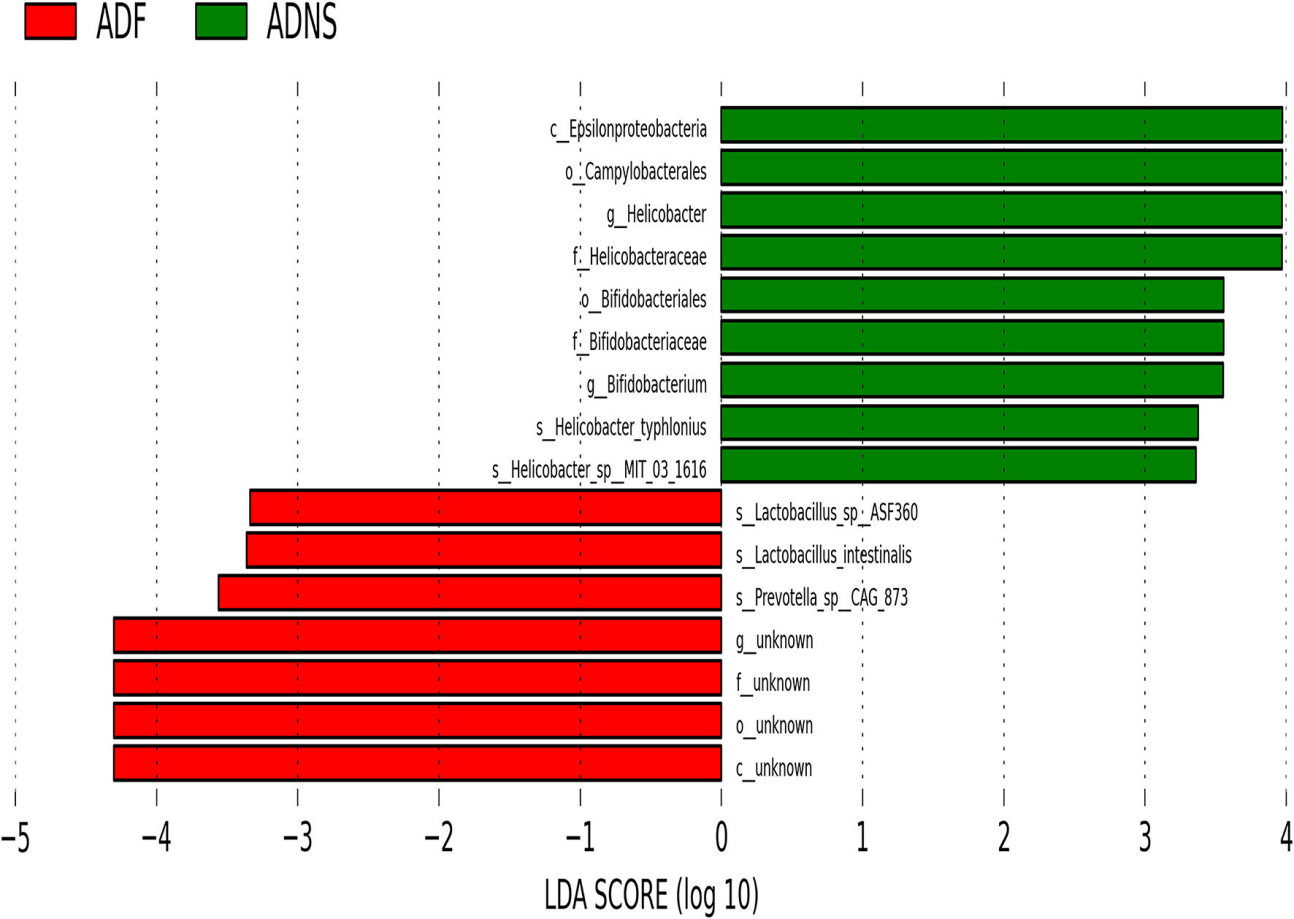
Linear discriminant analysis (LDA) effect size (LEfSe) analysis of microbiota in ADF (negative score) relative to ADNS (positive score). The LDA scores (log10) > ±3 were more abundant at the species level in ADNS compared to WT. *s_Prevotella_sp_CAG873* was identified as a potential biomarker in response to fasudil treatment in APP/PS1 mice (ADF), while *s_Helicobacter_typhlonius* and *s__Helicobacter_sp_MIT_03-1616* were identified as potential biomarkers in APP/PS1 mice (ADNS); *n* = 5.

The species levels of *s_Bacteroides_dorei_CAG222* (*p* < 0.05), *s_Bacteroidetes_bacterium_OLB8* (*p* < 0.05), *s_Prevotella_sp_CAG1031* (*p* < 0.05), and *s_Prevotella_sp_CAG873* (*p* < 0.01) in ADF were significantly higher as compared to ADNS, which exhibited significantly lower abundance as compared to WT (*p* < 0.05), suggesting that fasudil treatment reversed these species abundance (Figure 4 upper panels and Supplementary Table 3: the level of species in all groups). Similarly, the abundance levels of some species in ADNS were significantly lower as compared to WT, including *s_Alistipes_finegoldii* (*p* < 0.01), *s_Alistipes_sp_CAG53* (*p* < 0.05), *s_Alistipes_sp_CAG435* (*p* < 0.05), and *s_Butyricimonas_synergistica* (*p* < 0.01) (Figure 4 middle panels and Supplementary Table 4: the level of species in all groups). The abundance levels were increased after fasudil treatment, but with no significant difference between ADF and ADNS. In contrast, ADNS showed significantly more abundance in *s_Helicobacter_saguini, s_Helicobacter_typhlonius*, and *s_Helicobacter_sp_MIT_03-1616* compared to WT (*p* < 0.05) (Figure 4 lower panels and Supplementary Table 3: the level of species in all groups), which was reduced in ADF, with statistical significance in *s_Helicobacter_saguini* (*p* < 0.05), suggesting that fasudil treatment blocked AD-induced increases in the abundance of these species, especially *s_Helicobacter_saguini*. Finally, we used Lefse (LDA Effect Size, linear discriminant analysis) combined with the statistical analysis to screen key biomarkers (Liu et al., 2018, 2019). The LDA scores (log10 > ±3) indicates more abundance at the species level in ADNS and ADF are shown in Figure 5. The results of metagenomics demonstrated that *s_Prevotella_sp_CAG873* was identified as an ADF potential biomarker, while *s_Helicobacter_typhlonius* and *s_Helicobacter_sp_MIT_03_1616* were identified as ADNS potential biomarkers in the fecal of APP/PS1 mice (Figure 5).

### Fasudil Treatment Altered Gut Metabolites in APP/PS1 Mice

**Figure 6:**
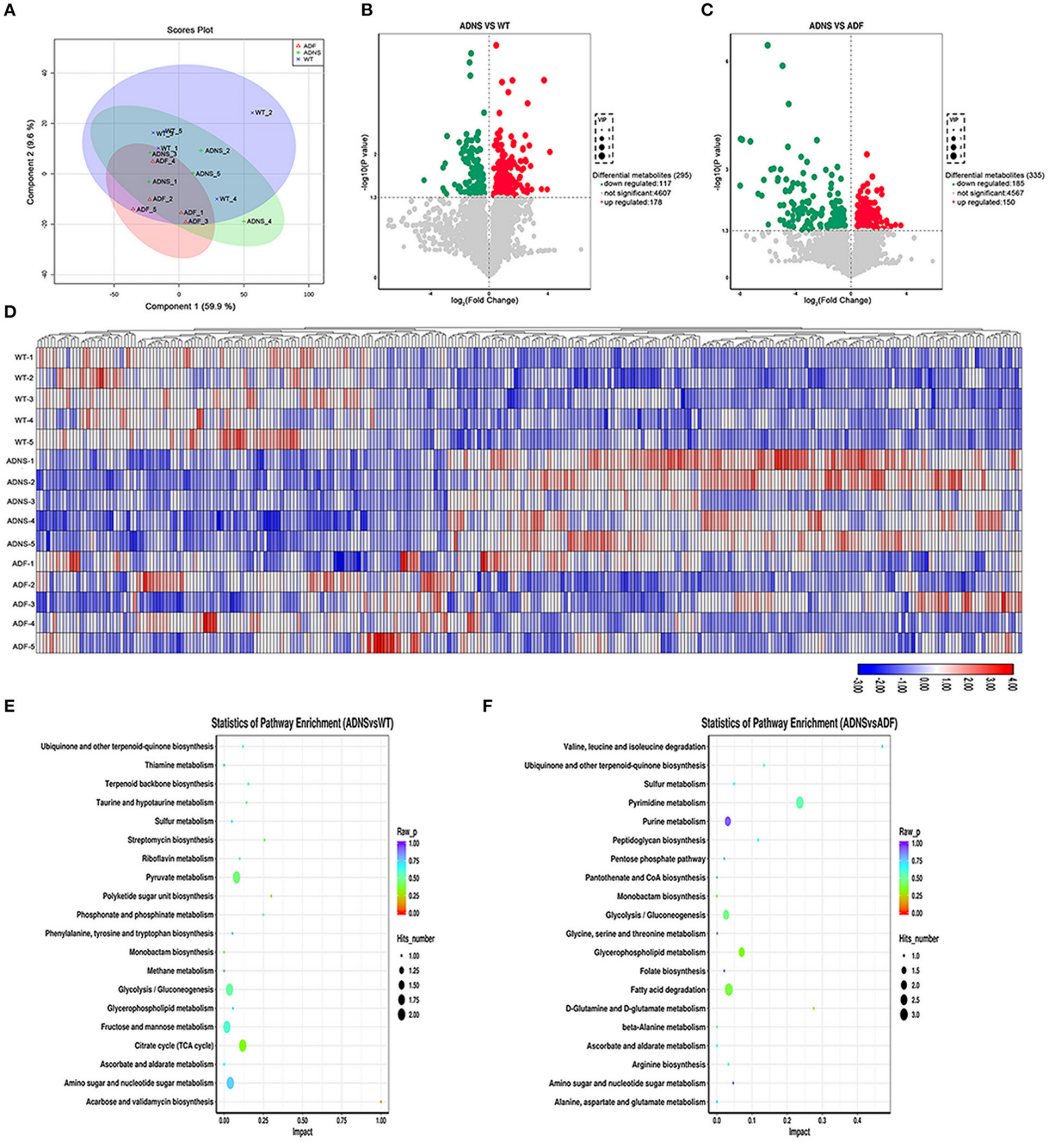
Intestinal metabolite alteration of APP/PS1 mice treated with fasudil. **(A)** The PLS-DA score plots of metabolic profiles in WT (blue) and APP/PS1 mice treated with saline (ADNS, in green), or fasudil (ADF, in red). The separation trend of metabolic changes was observed in ADNS, WT and ADF; all samples were analyzed with 95% confidence interval (CI). **(B,C)** Volcano diagram of the changes in metabolites in WT, ADNS, and ADF mice. There were 295 metabolites differentially expressed in ADNS vs WT **(B)**, including 117 metabolites significantly downregulated (green) and 178 metabolites significantly upregulated (red) (metabolites with non-significant differences are shown in gray). In addition, there were 335 metabolites differentially expressed in ADF vs. ADNS **(C)**, including 185 downregulated metabolites and 150 upregulated metabolites. Each point in the volcano diagram represents a single metabolite. The scatter color represents the final screening result. **(D)** The heat map by the Hierarchical Clustering Analysis for different comparison combinations with significant changes. ADNS (middle 1–5) was presented in a different color pattern relative to WT (upper 1–5), which was similar to ADF (lower 1–5). **(E,F)** Analysis of the top 20 metabolic pathways in comparison combinations according to the impact factors (bubble plot). The results of the metabolic pathway analysis were presented as bubble plots. The bubble color represents the p value of the enrichment analysis, while the size of the point represents the number of different metabolites enriched in the pathway. Compared to WT, ADNS was focus on the metabolisms of pyruvate, glycolysis/gluconeogenesis, fructose, mannose, citrate cycle (TCA cycle), amino sugar, and nucleotide sugar; compared to ADNS, ADF was focused on the metabolisms of pyrimidine, purine, glycolysis/gluconeogenesis, glycerophospholipid, and fatty acid degradation; *n* = 5.

To determine the effect of fasudil on gut metabolites changes associated with AD, we carried out metabolomic analysis using UHPLC-QTOFMS and PLS-DA cluster analysis and determine the trends of metabolic changes in ADNS, relative to WT or ADF. All samples were analyzed with a 95% confidence interval (Hotelling's t-squared ellipse; Figure 6A). The results from the screening of different metabolites were visualized in the form of volcano plots (Figures 6B,C). There were 295 different metabolites in ADNS vs. WT, including 117 decreased and 178 increased metabolites (Figure 6B); 335 different metabolites in ADNS vs. ADF, including 185 decreased and 150 increased metabolites (Figure 6C). Further clustering analysis of differential metabolites in each group revealed that ADNS had a different heat map from WT and ADF, both of which were presented similarly (Figure 6D). In addition, the 295 differential metabolites in ADNS vs. WT were enriched in 28 signaling pathways (Figure 6E and Supplementary Table 4: the differential metabolites in ADNS vs. WT were enriched in 28 signaling pathways), focused on the metabolisms of pyruvate, glycolysis/gluconeogenesis, fructose and mannose, citrate cycle (TCA cycle), amino sugar, and nucleotide sugar; the 335 differential metabolites in ADNS vs. ADF were concentrated in 20 signaling pathways focusing on the metabolisms of pyrimidine, purine, glycolysis/gluconeogenesis, glycerophospholipid, and fatty acid degradation (Figure 6F and Supplementary Table 5: the differential metabolites in ADNS vs. ADF were concentrated in 20 signaling pathways).

### Association Analysis of the Metagenomic and Metabolomic Profiles

To examine the correlation between metagenomic and multiple metabolites in AD, we analyzed the metabolites from gut microbiota and host in APP/PS1 mice. By taking the intersection of ADNS-WT and ADNS-ADF, 83 important, differential metabolites were obtained and narrowed down to 60 different metabolites by adjustment of *p* < 0.04 (Supplementary Table 6: the 60 differential metabolites by adjustment of *p* < 0.04), among which 20 important, different metabolites were screened as per available literatures [Supplementary Table 7: the correlation analysis of the species and 20 differential metabolites (p value)].

**Figure 7:**
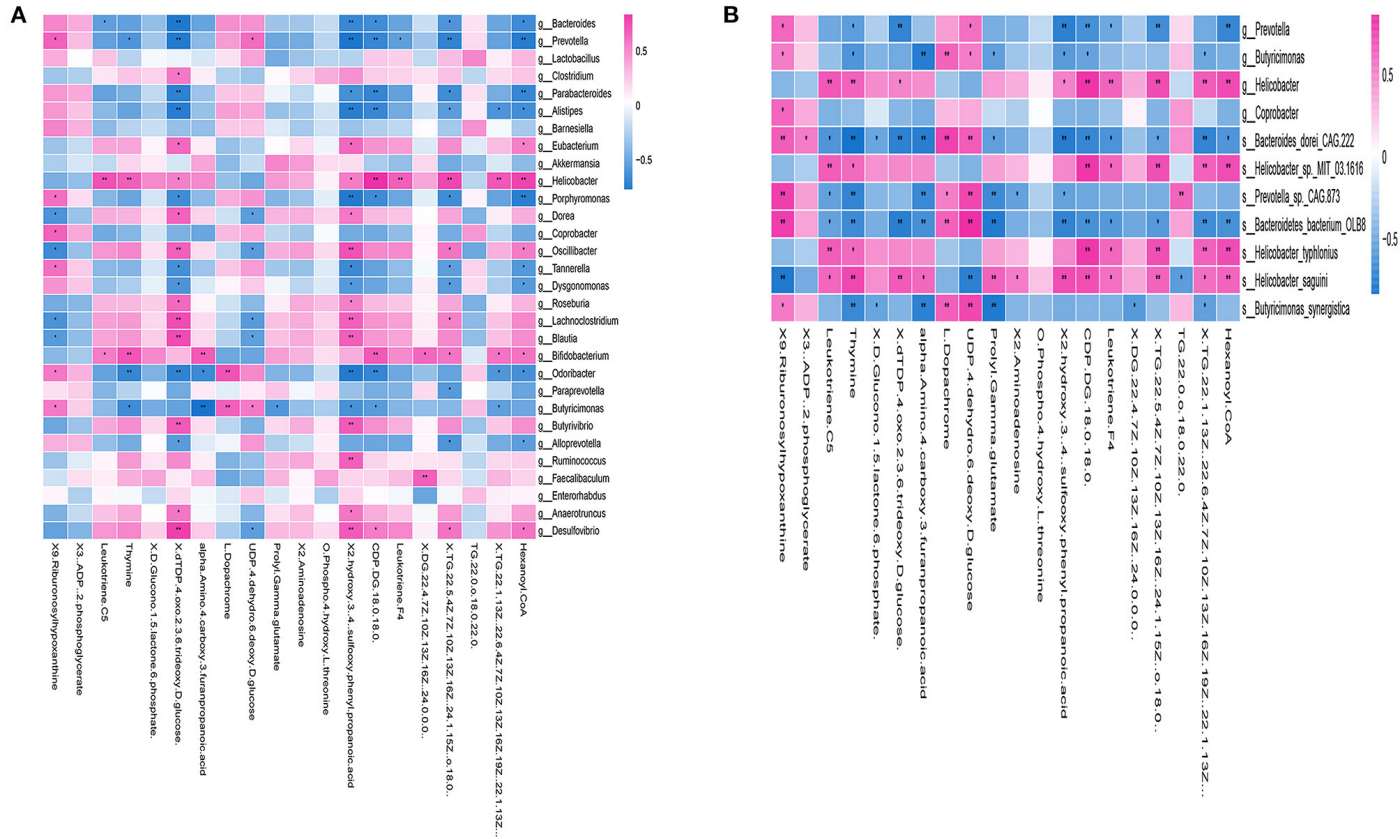
Correlation between gut microbiotas and metabolites. **(A)** Correlation analysis at the genus level of the 30 most abundant gut microbiotas and 20 different metabolites. Some microbiotas were correlated with specific metabolites, including *g_Clostridium*, which showed a positive correlation with dTDP-4-oxo-2,3,6-trideoxy-d-glucose (*p* < 0.05), and *g_Faecalibaculum*, which had a positive correlation with DG (22:4(7Z,10Z,13Z,16Z) (*p* < 0.01). **(B)** The heat map of the most abundant species and 20 different metabolites. The correlation analysis revealed that both of s_Bacteroides_dorei_CAG222 and s_Bacteroidetes_bacterium_OLB8 were correlated with 14 different metabolites, including 9-ribofuranosyl hypoxanthine, leukotriene C5, thymine, dTDP-4-oxo-2,3,6-trideoxy-D-glucose, alpha-amino-4-carboxy-3-furanpropanoic acid, L-dopachrome, UDP-4-dehydro-6-deoxy-D-glucose, prolyl-gamma-glutamate, 2-hydroxy-3-[4-(sulfooxy)phenyl]propanoic acid, CDP-DG(18:0/18:0), leukotriene F4, TG(22:5(4Z,7Z,10Z,13Z,16Z), TG(22:1(13Z)/22:6(4Z,7Z,10Z,13Z,16Z,19Z), and hexanoyl-CoA, all of which were widely related to a variety of metabolisms, such as carbohydrate metabolism and fatty acid metabolism. *p < 0.05, **p < 0.01; positive correlation is indicated in red and negative correlation is in blue; *n* = 5.

Correlation analysis of genus levels in top 30 abundance of gut microbiota and 20 different metabolites were presented in Figure 7A. Few gut microbiotas were correlated with a single metabolite, including *g_Clostridium*, which had a positive correlation with dTDP-4-oxo-2,3, 6-trideoxy-d-glucose (*p* < 0.05), and *g_Faecalibaculum*, showing a positive correlation with DG (22:4(7Z,10Z,13Z, 16Z)/24:0/0:0) (*p* < 0.01). Others were associated with a variety of metabolites [Supplementary Table 7: the correlation analysis of the species and 20 differential metabolites (p value)].

Furthermore, we examined 20 different metabolites correlation with the ADNS biomarkers (*s_Helicobacter_typhlonius, s_Helicobacter_sp_MIT_03-1616*) or the ADF biomarker (*s_Prevotella_sp_CAG873*). In addition, it was shown that s_*Bacteroides_dorei_CAG222* and *s_Bacteroidetes_bacterium_OLB8* were significantly lower in ADNS compared to WT; this was reversed in ADF (*p* < 0.05; Figure 4 upper; Supplementary Table 3: the level of species in all groups). The correlation analysis revealed that the two species were correlated with 14 metabolites, including 9-ribofuranosyl hypoxanthine, leukotriene C5, thymine, dTDP-4-oxo-2,3,6-trideoxy-D-glucose, alpha-amino-4-carboxy-3-furanpropanoic acid, L-dopachrome, UDP-4-dehydro-6-deoxy-D-glucose, prolyl-gamma-glutamate, 2-hydroxy-3-[4-(sulfooxy)phenyl]propanoic acid, CDP-DG(18:0/18:0), leukotriene F4, TG(22:5(4Z,7Z,10Z,13Z,16Z), TG(22:1(13Z)/22:6(4Z,7Z,10Z,13Z,16Z,19Z), and hexanoyl-CoA, which were widely related to a variety of metabolisms, such as carbohydrate metabolism and fatty acid metabolism. [Figure 7B, Supplementary Table 7: the correlation analysis of the species and 20 differential metabolites (p value)].

A heatmap was used to show the correlation results in Figure 7B, which showed a specific positive correlation between the ADNS biomarkers (*s_Helicobacter_typhlonius, s_Helicobacter_sp_MIT_03-1616*), and 5 metabolites, including leukotriene F4, CDP-DG (18:0/18:0), TG (22:5 (4Z,7Z,10Z,13Z,16Z), TG (22:1(13Z)/22:6 (4Z,7Z,10Z,13Z,16Z,19Z), hexanoyl-CoA (*p* < 0.01). All the 5 metabolites were significantly higher in ADNS than in WT, significantly lower in ADF than in ADNS, and returned to the WT levels in ADF [Supplementary Table 7: the correlation analysis of the species and 20 differential metabolites (p value)]. These 5 metabolites are mainly involved in lipid metabolism, indicating that fasudil might protect neurons by decreasing hexanoyl-CoA, a short-chain fatty acyl-CoA precursor. Notably, fasudil also significantly decreased leukotriene F4, which is involved in the proinflammatory pathways.

There were 8 metabolites specifically correlated with the ADF biomarker (*s-Prevotella sp_CAG873*), 4 of which were significantly higher in ADNS compared to WT or ADF, including alpha-amino-4-carboxy-3-furanpropanoic acid, prolyl-gamma-glutamate, 2-aminoadenosine, and 2-hydroxy-3-[4-(sulfooxy) phenyl] propanoic acid; in other word, ADF reduced ADNS-induced high contents of these metabolites to the WT levels (Supplementary Table 8). The other four metabolites were significantly lower in ADNS relative to WT or ADF, including 9-Ribofuranosyl hypoxanthine, L-dopachrome, UDP-4-dehydro-6-deoxy-D-glucose, and TG (22:0/o-18:0/22:0); in other words, ADF increased these metabolites in ADNS to the WT levels [Supplementary Table 7: the correlation analysis of the species and 20 differential metabolites (p value)]. The 8 metabolites were highly related to carbohydrate, nucleotide, and fatty acid metabolism.

It was noted that two of the metabolites, i.e., leukotriene C5 and thymine, were correlated with both the ADF biomarker (*s_Prevotella sp_CAG873*) and the ADNS biomarkers (*s_Helicobacter_typhlonius, s_Helicobacter_sp_MIT_03-1616*). They were significantly higher in ADNS compared to WT or ADF; in other words, ADF decreased the two metabolites in ADNS to the WT levels [Figure 7B, Supplementary Table 7: the correlation analysis of the species and 20 differential metabolites (p value)].

### Functional Annotations of Association Metabolites With Genes of Biomarkers of ADF or ADNS

**Figure 8:**
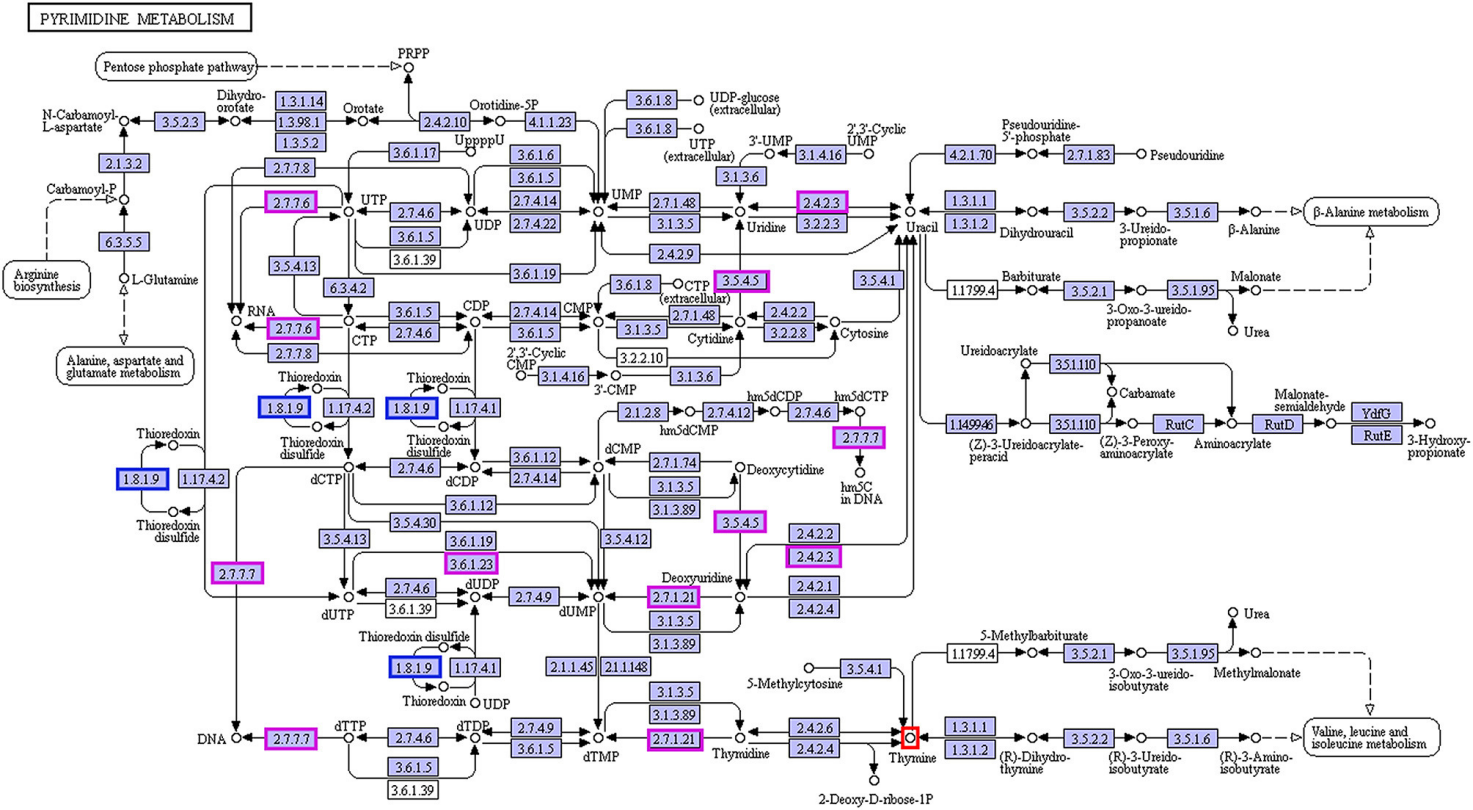
Correlation between thymine and the biomarkers for ADF and ADNS. The enzyme EC:1.8.1.9 (in blue rectangles) encoded by the gene trxB was correlated with the ADNS biomarker (*s_Helicobacter_sp_MIT_03-1616*), while the enzymes EC:2.7.7.6, EC:3.5.4.5, EC:2.7.1.21, EC:2.7.7.7, EC:3.6.1.23, and EC:2.4.2.3 (in pink rectangles) were encoded by the genes rpoC, cdd/CDA, tdk/TK, holA /DPO3D1, dut/DUT, udp/UPP, respectively; they were correlated with in the ADF biomarker (s-Prevotella sp CAG873) (also see Table 1 for the genes), *n* = 5. EC:1.8.1.9, thioredoxin-disulfide reductase; EC:2.7.7.6, DNA-directed RNA polymerase; EC:3.5.4.5, cytidine deaminase; EC:2.7.1.21, thymidine kinase; EC:2.7.7.7, DNA-directed DNA polymerase; EC:3.6.1.23, dUTP diphosphatase; EC:2.4.2.3: uridine phosphorylase; trxB, thioredoxin reductase gene; rpoC, RNA polymerase subunit C gene; cdd, cytidine deaminase gene; tdk, the thymidine kinase gene; holA, holA gene encoded one subunit of DNA polymerase III holoenzyme; dut, dUTPase (DUT) gene; udp, uridine diphosphate gene.

In the enrichment analysis of functional annotations of association of metabolites with genes of biomarkers of ADF or ADNS, we observed significant pathways mainly involved in amino sugar, nucleotide sugar, pyrimidine metabolisms. As shown in Table 1, UDP-4-dehydro-6-deoxy-D-glucose was correlated with the ADF biomarker (*s_Prevotella sp_CAG873*), while thymine was correlated with both the ADF biomarker (*s_Prevotella sp_CAG873*) and the ADNS biomarker (*s_Helicobacter_sp_MIT_03-1616*). Both the ADNS and ADF biomarkers contained genes involved in signaling pathways of the pyrimidine metabolism (ko00240), encoded various enzymes, and further influenced the production of thymine. More specifically, the thioredoxin reductase gene (trxB) in the ADNS biomarker (*s_Helicobacter_sp_MIT_03-1616*) encoded the enzyme thioredoxin-disulfide reductase (EC:1.8.1.9) (blue rectangles, Figure 8; Table 1), likely to affect the refolding of post-translation protein. In the ADF biomarker (*s-Prevotella sp CAG873*), genes such as the RNA polymerase subunit C gene (rpoC), cytidine deaminase gene (cdd, CDA), thymidine kinase gene (tdk), holA gene-encoded one subunit of DNA polymerase III holoenzyme (holA), dUTPase gene (dut), and uridine diphosphate gene(udp), respectively encoded the following enzymes: DNA-directed RNA polymerase (EC:2.7.7.6), cytidine deaminase (EC:3.5.4.5), thymidine kinase (EC:2.7.1.21), DNA-directed DNA polymerase (EC:2.7.7.7), dUTP diphosphatase (EC:3.6.1.23), and uridine phosphorylase (EC:2.4.2.3) (pink rectangles, Figure 8; Table 1).

**Table 1:** Functional annotations of associated metabolites with genes of biomarkers of ADF or ADNS mice.

**Name**	**KO_id**	**Gene_name**	**EC**	**Metabolites**
s_Prevotella sp. CAG:873 ko00520 (Amino sugar and nucleotide sugar metabolism)	K07106	murQ	EC:4.2.1.126	UDP-4-dehydro-6-deoxy-D-glucose
	K01835	pgm	EC:5.4.2.2	
	K12373	HEXA_B	EC:3.2.1.52	
	K02472	wecC	EC:1.1.1.336	
	K01443	nagA, AMDHD2	EC:3.5.1.25	
s__Prevotella sp. CAG:873 ko00240 (Pyrimidine metabolism)	K03046	rpoC	EC:2.7.7.6	Thymine
	K01489	cdd, CDA	EC:3.5.4.5	
	K00857	tdk, TK	EC:2.7.1.21	
	K02340	holA, DPO3D1	EC:2.7.7.7	
	K01520	dut, DUT	EC:3.6.1.23	
	K00757	udp, UPP	EC:2.4.2.3	
s_Helicobacter sp. MIT 03-1616 ko00240 (Pyrimidine metabolism)	K00384	trxB	EC:1.8.1.9	Thymine

## Discussion

**Figure 9:**
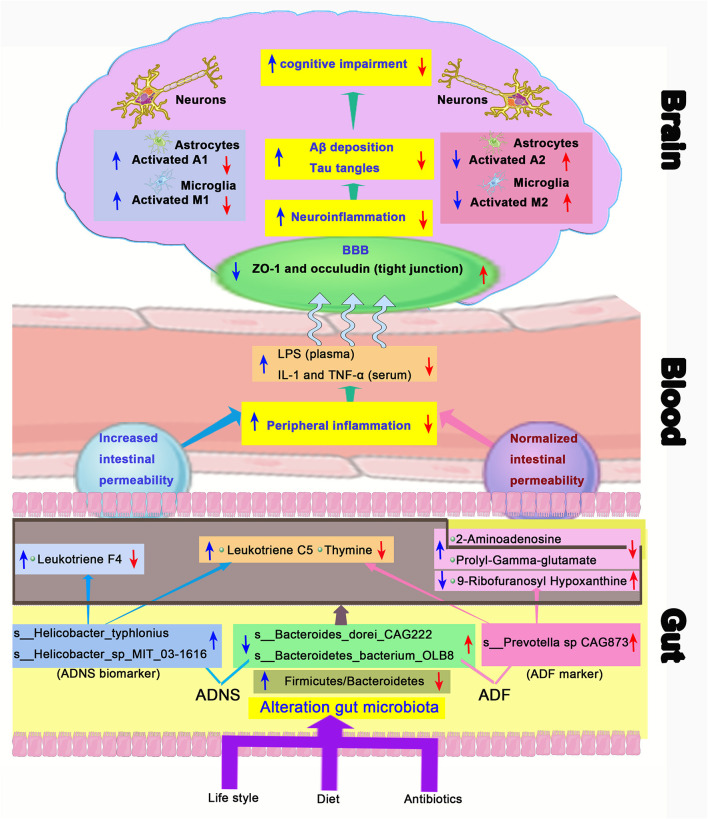
Schematic diagram of the gut-brain axis in AD treated with or without fasudil. Changes in the gut microbiota in the mouse model of AD (ADNS) cause abnormal production of metabolites, which aggravate peripheral inflammation, leading to increases in the brain infiltration of immune cells. Microglia M1 and astrocytes A1 cells are then activated in the brain, resulting in Aβ deposition, tau phosphorylation, and cognitive impairment. Treatment with fasudil (ADF) reconditions the gut microbiota, normalizes disordered metabolites, reduces the peripheral immune cell infiltration to the brain, ameliorates neuroinflammation, and lowers the accumulation of Aβ deposition and pTau, leading to ultimate improvement of cognitive functions. Blue arrows represent ADNS-related changes, while the red arrows refer to ADF-related changes. BBB, blood brain barrier; LPS, lipopolysaccharide; IL-1, interleukin-1; TNF-α, tumor necrosis factor -α; ZO-1, zonula occluden-1.

Accumulating clinical studies support that Aβ and pTau acts as important biomarkers of AD (Blennow and Zetterberg, 2018; Jack et al., 2018; Fuller et al., 2019). It has been demonstrated that alteration of gut microbial community is associated with AD and gut microbiota are changed even before the onset of AD (Li et al., 2019). Diet plays an important role in determining the composition of the gut microbiota, which is known to impact metabolic functions as well as immune responses (Anand and Mande, 2018). A specific anti-AD therapy is usually combined with lifestyle interventions (Scheltens et al., 2016) (Figure 9). In the present study, we demonstrated the amelioration of impairment of learning and memory and revealed the relationship between the gut microbiome and metabolites in APP/PS1 mice following treatment with the ROCK inhibitor fasudil.

Clinical studies have shown that patients with *Helicobacter pylori* infection exhibit symptoms of AD. *Helicobacter pylori* crosses the blood-brain barrier (BBB) and contribute to amyloid deposition (Contaldi et al., 2017; Beydoun et al., 2020), damages the BBB and subsequently cause direct interactions of gut metabolites with enteric neurons in the brain (van de Haar et al., 2015). Microbial metabolites also influence the peripheral immune response, consequently affects the BBB (Cryan and Dinan, 2012). Neurofibrillary tangles induced by Aβ and pTau causes blood vessel abnormalities and BBB breakdown, which contribute to cognitive decline in APOE4 carriers independent of AD pathology (Montagne et al., 2020). Fasudil retains the BBB integrity by up-regulating expression of tight junction proteins ZO-1 and occludin as demonstrated in our previous studies (Kubo et al., 2008; Huang et al., 2011; Fujii et al., 2012; Niego et al., 2017; Yan et al., 2019). Therapeutic effects of fasudil could be due to prevention of intestinal mucosal barrier disruption, decreased release of amyloid peptides and lipopolysaccharides (LPS), and inactivation of inflammatory signaling induced by cytokines (de and Forlenza AS, 2018; Doulberis et al., 2021).

Glutamatergic neurons in the hippocampus are closely associated with AD pathogenesis (Danysz and Parsons, 2012; Moon et al., 2019). Abnormal increases in glutamate, an important excitatory neurotransmitter in neurons, can cause cell death (Zhang et al., 2016). Understanding the molecular mechanism of glutamate would help to develop novel and effective targets for AD treatment (Pereira et al., 2017). Anti-glutamatergic drugs such as memantine has been shown to exhibit beneficial effects in AD treatment (Zott et al., 2019). Aβ-induced cognitive impairment has been proven to be mediated by glutamatergic neurons (Findley et al., 2019). Consistent with reported studies, our study showed that fasudil treatment reduced prolyl-gamma-glutamate and improved cognition in APP/PS1 mice.

9-ribofuranosyl hypoxanthine was significantly reduced, while thymine was incresaed in ADNS as compared to WT or ADF, i.e., both changes were reversed by fasudil treatment. This is supported by the finding that hypoxanthine, a purine derivative and a product of DNA metabolism following apoptosis and cell lysis (Chouraki et al., 2017), is decreased at the early stages of AD-related pathology (Alonso-Andres et al., 2018). Deficiency of hypoxanthine-guanine phosphoribosyltransferase (HGPRT) re-directs gene expression from neuronal to glial functions (Kang and Friedmann, 2015), leading to increased microglia-mediated neuroinflammatory responses that contributes to AD-associated neurodegeneration (Markesbery and Lovell, 2006; Grathwohl et al., 2009). Fasudil has been shown to inhibit macrophages/microglia (M1) and astrocytes (A1), while increased expression of macrophages/microglia (M2) and astrocytes (A2) in the peripheral and central immune systems (Wang et al., 2019) (Figure 9). Thymine is related to metabolism of isoleucine, which promotes both differentiation and proliferation of peripheral pro-inflammatory T helper 1 (Th1) cells (Wang et al., 2019).

Leukotrienes are a family of major pro-inflammatory lipid mediators produced in leukocytes by the lipoxygenase (LOX) pathway of the arachidonic acid metabolism (Rahman et al., 2019). Over-activation and/or overexpression of 5-LOX in AD are typically indicative of an inflammatory basis for AD pathobiology (Manev et al., 2011). The leukotriene pathway can be a potential target to reduce the inflammation and ameliorate various aspects of AD pathology (Michael et al., 2019). There is restoration of homeostasis and promotion of tissue healing by increasing anti-inflammatory cytokine levels and decreasing pro-inflammatory mediators (Whittington et al., 2017). These support our data that fasudil decreased leukotrienes in AD mice, which showed increases in leukotrienes and hexanoyl-CoA. Fasudil also decreased hexanoyl-CoA in AD mice. It is not clear whether AD is mediated by hexanoyl-CoA, which is the main factor in the synthesis of cannabinoids and is associated with allergies. Hexanoyl-CoA is one of the potential target molecules for intervention of spleen deficiency syndrome (Stout et al., 2012).

Alteration of cerebral glucose metabolism plays vital role in pathogenesis of AD (Chen and Zhong, 2013). Brain glucose uptake is impaired in AD (Cunnane et al., 2016). The disruption of homeostasis in lipid and glucose metabolism might aggravate neurodegeneration and/or cognitive dysfunction through the accumulation of Aβ and pTau and/or by impairing of neuronal integrity (Shinohara and Sato, 2019). High glucose concentrations-induced reduction of glycolytic flux in the brain is associated with the severity of AD pathology (An et al., 2018). UDP-4-dehydro-6-deoxy-D-glucose is mainly generated by glycolysis and participates in the N-glycan biosynthesis process (Schwetz et al., 2010). Increased activities of glucose-6-phosphate dehydrogenase have been found in AD tissues due to elevated brain peroxide metabolism (Martins et al., 1986). It is consistent with our results that UDP-4-dehydro-6-deoxy-D-glucose was significantly decreased in AD mice as compared to WT and was returned to the WT level after treatment with fasudil.

## Limitation

The data were generated from APP/PS1 mice. While this is translatable, the conclusions need to be verified in clinic studies in the future.

## Conclusions

The present study demonstrated that the gut microbiota composition and metabolites were altered in APP/PS1 mice. Fasudil reversed the abnormal gut microbiota and subsequently regulated the related metabolisms to normal in the AD mice. Modulation of gut microbiotas through personalized diet or beneficial microbiota intervention may be a potential preventive treatment strategy for AD. The alteration of the gut microbiota composition caused changes in microbiome state, further influencing the pathway of lipid metabolism, amino acid metabolism, glucose metabolism, and nucleotide metabolism. Therefore, fasudil may be a novel strategy for the treatment of AD via remodeling of the gut microbiota and metabolites. The novel results also provide valuable references for the use of gut microbiota and metabolites as diagnostic biomarkers and/or therapeutic targets in clinical studies of AD.

## Data Availability Statement

The datasets presented in this study can be found in online repositories. The names of the repository/repositories and accession number(s) can be found below: NCBI (Microbiota ID:PRJNA754523; Metabolites ID:PRJNA754682).

## Ethics Statement

The animal study was reviewed and approved by Animal Ethics Committee of Shanxi Datong University, Datong, China.

## Author Contributions

YY and YG designed the study, carried out the animal tests, data analysis, outlined, and drafted the manuscript. GK and KS helped in revision the manuscript. QF, NZ, HY, and LS participated in animal tests and data analysis. YY, YG, GK, H-TZ, and C-GM revised and finalized the manuscript. JL, YZ, JS, JW, and LZ performed the animal treatment experiments. All authors read and approved the final manuscript.

## Funding

This work was supported by research grants from the National Natural Science Foundation of China (No. 81473577 to C-GM) and the Department of Science and Technology, Shanxi Province of China (201803D421073 to YY, 201805D111009 to C-GM) for the sample analysis. Science and Technology Innovation Project of Universities, Shanxi Province of China (2019L0765 to YG), Datong Municipal Science and Technology Bureau (2019198 to C-GM and YY, 2020061 to HY) for animal treatment. Leading Team of Medical Science and Technology, Shanxi Province (2020TD05 to C-GM) and Young Scientists Cultivation Project of Shanxi University of Chinese Medicine (2021PY-QN-09 to LS) for and Ph.D. Initiation Grant of Datong University (2019-B-07 to HY) for research organization.

## Conflict of Interest

The authors declare that the research was conducted in the absence of any commercial or financial relationships that could be construed as a potential conflict of interest.

## Publisher's Note

All claims expressed in this article are solely those of the authors and do not necessarily represent those of their affiliated organizations, or those of the publisher, the editors and the reviewers. Any product that may be evaluated in this article, or claim that may be made by its manufacturer, is not guaranteed or endorsed by the publisher.

## Supplementary Material

Supplementary Table 1 Dominant phyla in all groups.

Supplementary Table 2 Level of family in all groups file.

Supplementary Table 3 Level of species in all groups file.

Supplementary Table 4 Differential metabolites in ADNS vs. WT were enriched in 28 signaling pathways.

Supplementary Table 5 Differential metabolites in ADNS vs. ADF were concentrated in 20 signaling pathways.

Supplementary Table 6 60 differential metabolites by adjustment of p£° 0.04.

Supplementary Table 7 Correlation analysis of the species and 20 differential metabolites (p value).
