# Supplementary figures and images for: Inhibition of Rho Kinase by Fasudil Ameliorates Cognition Impairment in APP/PS1 Transgenic Mice via Modulation of Gut Microbiota and Metabolites
Source: Front Aging Neurosci. 2021 Oct 14;13:755164. doi: 10.3389/fnagi.2021.755164 (PMC8551711; doi:10.3389/fnagi.2021.755164)

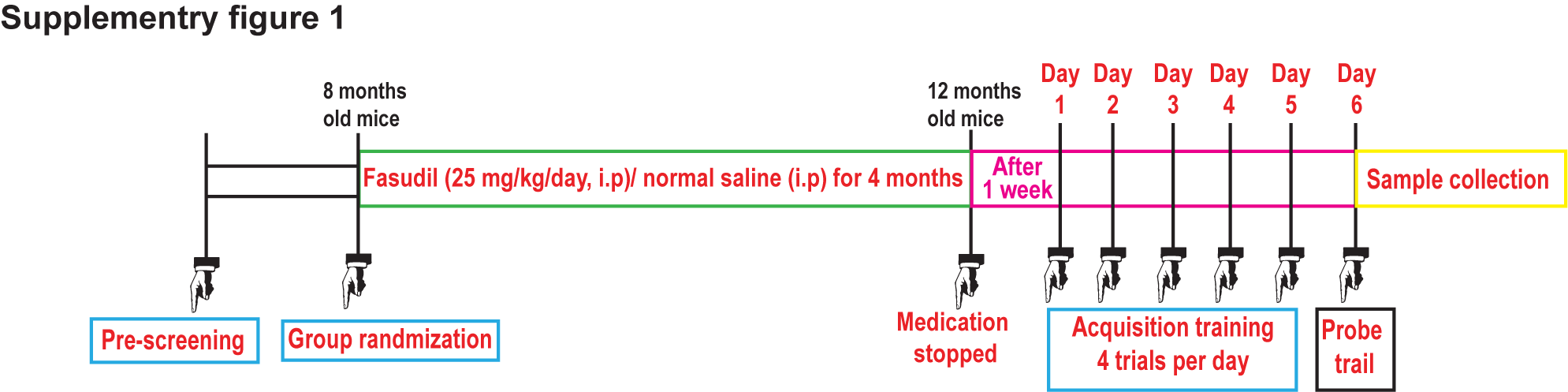

Supplement: Supplementary file 8 [file Image_1.tif]
